# A retrospective comparative analysis of elderly and younger patients undergoing pulmonary resection for stage I non-small cell lung cancer

**DOI:** 10.1186/s12957-015-0762-8

**Published:** 2016-01-19

**Authors:** Byungjoon Park, Genehee Lee, Hong Kwan Kim, Yong Soo Choi, Jae Il Zo, Young Mog Shim, Jhingook Kim

**Affiliations:** Department of Thoracic and Cardiovascular Surgery, Samsung Medical Center, Sungkyunkwan University School of Medicine, 81 Irwon-ro, Gangnam-gu, Seoul, 135-710 South Korea

**Keywords:** Lung cancer, Surgery, Geriatric, Pulmonary function

## Abstract

**Background:**

Age has been a critical predictor for immediate postoperative and long-term results after the pulmonary resection for lung cancer. In this study, we evaluated and compared surgical outcome of stage I non-small cell lung cancer and associated predictive factors between elderly and younger groups.

**Methods:**

Short- and long-term outcomes of elderly group (≥70 years) who were surgically treated and pathologically diagnosed as stage I non-small cell lung cancer from 2004 to 2010 were compared to the results of younger group (<70 years).

**Results:**

Total of 1340 patients were included in this study, and the patients were divided into the elderly group (*n* = 285) and the younger group (*n* = 1055). The proportions of squamous cell carcinoma (36.8 vs. 20.0 %, *p* < 0.001) and stage IB cancer (58.3 vs. 40.6 %, *p* < 0.001) were significantly higher in the elderly group than the younger group. The 30-day and 90-day mortalities were significantly higher in the elderly group (1.8 vs. 0%; *p* = 0.014, 3.9 vs. 0.5 %; *p* < 0.001, respectively). The elderly patients also had significantly worse long-term outcomes than the younger group (5-year overall survival rate, 69.0 vs. 91.1 %; *p* < 0.001, 5-year disease-free survival rate, 53.3 vs. 80.2 %; *p* < 0.001). Decreased diffusion capacity less than 70 % was an important predictive factor for short- and long-term outcomes in both the younger and the elderly group.

**Conclusions:**

Elderly patients with low diffusion capacity are at risk for significantly worse outcome, indicating that patient selection should include assessment of pulmonary function, including diffusion capacity.

## Background

Lung cancer is a dreadful disease, but better results can be obtained with early detection and effective surgery. Accordingly, considerable effort has been devoted to early diagnosis of lung cancer. Lung cancer is the second most common cancer for those aged 70 years or older, and the likelihood of developing new lung cancer was 1 in 15 for males and 1 in 20 for females in the USA in 2013 [1]. Rapid expansion of the geriatric population in both developed and developing countries has increased the number of cases of newly diagnosed lung cancer in elderly patients, and lung cancer has literally become a “disease of elderly people” [2].

However, surgical treatment, the gold standard in early lung cancer, is not always recommended for elderly patients compared to younger patients. According to the previous studies, only less than 50 % of patients older than 75 years were rendered to undergo surgical resection even for early non-small cell lung cancer (NSCLC) for a variety of reasons, including comorbidities and personal decisions not to pursue further treatment [3, 4]. On the other hand, a few encouraging results of successful surgery in octogenarians or nonagenarians with early lung cancer have been reported [5–7]. Even so, most existing studies were conducted in highly selected patient populations and focused mainly on early outcomes, making them insufficient to justify recommending for or against surgery in elderly patients in general.

To confirm that pulmonary resections in elderly patients are as safe as in younger patients, it is important to compare surgical outcomes and also associated predictors. Furthermore, if elderly patients show worse outcomes, it is important to identify not only common predictive factors in both age groups but also age-specific factors, and this would be helpful to establish more elderly-specific guideline for selecting surgery patients. In this study, we evaluated and compared short- and long-term outcomes of elderly and younger patients who underwent pulmonary resection for stage I NSCLC and analyzed common predictive factors and specific factors for both age groups.

## Methods

### Patients and data collection

This was a retrospective observational study performed in a single center and approved by the hospital’s institutional review board. From January 2004 to December 2010, 3033 patients were surgically treated with curative intent for primary lung cancer, and 1787 patients were pathologically diagnosed with stage I NSCLC. Patients with synchronous double primary lung cancer (*n* = 49), previous history of other malignancy (*n* = 162) and those without mediastinal lymph node dissection (*n* = 145) were excluded from the study. Patients with histologic subtypes other than adenocarcinoma, squamous cell carcinoma, or large cell neuroendocrine carcinoma were also excluded (*n* = 107).

A total of 1340 patients were enrolled, with 285 patients (21.3 %) in the elderly group (≥70 years) and 1055 patients (78.7 %) in the younger group (<70 years). Significant cutoff value of age groups by 5 years for 30-day mortality and overall survival was analyzed by a minimal *p* value approach. Data on clinical, surgical, and pathologic characteristics were collected. Preoperative pulmonary function tests included forced expiratory volume in 1 second (FEV_1_), forced vital capacity (FVC), FEV_1_/FVC, percentile expected FEV_1_, percentile expected FVC, diffusing capacity for carbon monoxide (DLCO), and percentile expected DLCO. Laboratory data including liver and kidney function tests and ejection fraction from preoperative echocardiography were also collected. The type of operation and extent of pulmonary resection were included as surgical factors.

### Surgical procedures and follow-up

The standard treatment for stage I NSCLC was lobectomy or bi-lobectomy, in patients with limited pulmonary function or severe emphysema, segmentectomy or wedge resection was performed. After surgery, 4 patients (1.4 %) from elderly group and 16 patients (1.5 %) from younger group underwent adjuvant chemotherapy. The patients visited the outpatient clinic and underwent computed tomography or positron emission tomography/computed tomography every 3 months for 2 years after the surgery and then every 6 months for the next 3 years. The patients visited the hospital annually after 5 years from the initial operation. The median follow-up period was 47.6 months.

Patient survival was reviewed, and the end date was defined as the date of latest follow-up or death. The disease-free survival period was defined as the interval from surgery to the first interpretation of recurrence in an imaging study. Cancer recurrence was defined as carcinoma recurring in the lung or a distant organ and was classified as loco-regional failure or distant failure according to the initial site of recurrence. In detail, local failure was defined as disease recurrence at the surgical resection margin, regional failure as tumor recurrence in the mediastinal, hilar, or supraclavicular lymph nodes, and lung cancer in the ipsilateral lung, pleura, or chest wall. Distant failure was defined as lung cancer other than loco-regional failure or cancer metastasis in a distant organ. Metachronous double primary cancer was defined according to modified Martini’s criteria and was excluded from cancer recurrence. Newly appeared pure ground glass opacity in the ipsilateral lung within 2 years was also excluded from cancer recurrence.

The date of death for patients who expired during the follow-up period was confirmed by our hospital (*n* = 64) or National Cancer Registration Statistics (*n* = 89). Living patients were followed-up in an outpatient clinic (*n* = 1163) or by telephone interview (*n* = 24) within 1 year of data collection.

### Statistical analyses

All data were statistically analyzed using STATA version 10 (2007, Stata Statistical Software: release 10; StataCorp LP, College Station, TX). Ninety-five percent confidence intervals corresponding to a 5 % significance level were used. Mean values were compared using a Student *t* test and median values by the Wilcoxon signed-rank test. Data were interpreted in two categories. Short-term results, including 30-day and 90-day mortality, hospital stay, in-hospital complication rate, and associated risk factors, were evaluated by logistic regression model, and there were no censored data during the follow-up period of 90 days. Long-term results, including 5-year overall and 5-year disease-free survival and 3-year cumulative recurrence rate, were analyzed with Kaplan-Meier and log-rank tests. Subgroup analyses for risk factors and prognostic factors were evaluated in the elderly group using Cox hazard modeling. For multivariate analyses, backward selection of variables was performed. Variables with significance level >0.1 were eliminated from the methods.

## Results

### Patient characteristics

Median age of elderly group was 73 years (70–86) and of younger group was 58 years (20–69). Male predominance (*p* = 0.003) and the proportions of squamous cell carcinoma (36.8 % vs. 20.0 %, *p* < 0.001) and stage IB cancer (58.3 vs. 40.6 %, *p* < 0.001) were significantly higher in the elderly group than the younger group. There was no significant difference in extent of pulmonary resection between the two groups, but open thoracotomy was more frequently performed in elderly patients (55.4 vs. 37.8 %, *p* < 0.001). Comparison of clinicopathologic features and surgical procedures were demonstrated in Table 1.

**Table 1:** Patient clinicopathologic features and surgical procedures

	Elderly group (*n* = 285)	Younger group (*n* = 1055)	*p*
Median age, years (range)	73 (70–86)	58 (20–69)	<0.001*
Sex, male, *n* (%)	201 (70.5)	642 (60.9)	0.003**
Pathologic stage, *n* (%)			<0.001**
Stage IA	119 (41.7)	527 (59.4)	
Stage IB	166 (58.3)	428 (40.6)	
Histologic subtype, *n* (%)			<0.001**
Adenocarcinoma	171 (60)	824 (78.1)	
Squamous cell carcinoma	105 (36.8)	211 (20)	
Large cell neuroendocrine carcinoma	9 (3.2)	20 (1.9)	
Extent of resection, *n* (%)			0.280**
Sublobar resection	23 (8.1)	82 (7.8)	
Lobectomy	250 (87.7)	947 (89.7)	
Bilobectomy	12 (4.2)	26 (2.5)	
Type of operation, *n* (%)			<0.001**
Video-assisted thoracoscopic surgery	127 (44.6)	656 (62.2)	
Thoracotomy	158 (55.4)	399 (37.8)	

*p value estimated by the Wilcoxon signed-rank test

**p value estimated by Pearson’s chi-square test

### Short-term results

The 30-day mortality rate was 6 of 1340 patients (0.4 %), and all were elderly. Five patients died of acute respiratory distress syndrome (ARDS) and one from postoperative pneumonia. The only independent factor for 30-day mortality in the elderly group was DLCO less than 70 % of prediction (low DLCO; odd ratio (OR) = 17.1, *p* = 0.024). Mortality within 90 days occurred for 16 patients (1.2 %) and was significantly more frequent in the elderly group (3.9 vs. 0.5 %, *p* < 0.001). Independent risk factors for 90-day mortality included being elderly (OR = 2.0, *p* = 0.042) and low DLCO (OR = 3.9, *p* < 0.001). The elderly group had significantly longer hospital stays (11.2 vs. 8.0 days, *p* < 0.001). The in-hospital complication rate was also significantly higher in elderly patients (47.7 vs. 26.9 %, *p* < 0.001). The most common major complications in the elderly group were new onset of arrhythmia requiring medicine (16.3 %) and acute lung injury or ARDS (5.7 %), and independent risk factors were low DLCO (OR = 3.5, *p* = 0.045) and male gender (OR = 7.8, *p* < 0.001).

### Long-term results

**Fig. 1:**
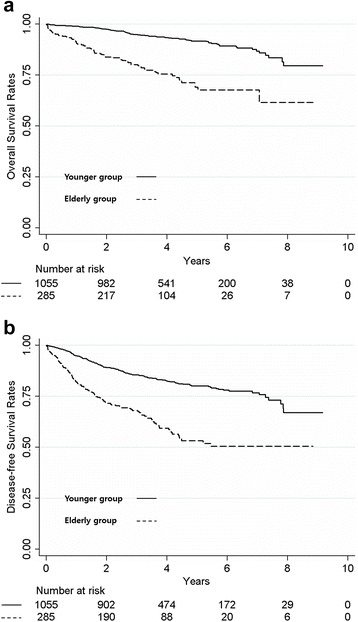
Kaplan-Meier curve of overall and disease-free survival in younger and elderly groups. **a** Five-year overall survival rates in the younger and elderly groups were 91.1 and 69.0 %, respectively. **b** Five-year disease-free survival rates in the younger and elderly groups were 80.2 and 49.3 %, respectively

Undoubtedly, elderly patients had significantly worse long-term outcomes than the younger group (5-year overall survival rate; 69.0 vs. 91.1 %, *p* < 0.001, Fig. 1). Even so, the difference between the two groups was much larger than expected. In the multivariate analysis, independent prognostic factors for overall survival after pulmonary resection of stage I NSCLC included elderly age (hazard ratio (HR) = 3.6, *p* < 0.001), male gender (HR = 3.2, *p* = 0.005), interstitial pulmonary fibrosis (IPF; HR = 4.8, *p* < 0.001), and low DLCO (HR = 3.8, *p* < 0.001). Prognostic factors for overall survival were summarized in Table 2. For the subgroup analysis in the elderly group, independent prognostic factors for overall survival included histologic subtype of squamous cell carcinoma (HR = 3.2, *p* = 0.001) and low DLCO (HR = 2.4, *p* = 0.028). On the other hand, independent prognostic factors for overall survival in the younger group were low DLCO (HR = 5.3, *p* = 0.004), male gender (HR = 5.3, *p* = 0.026), and IPF (HR = 14.1, *p* < 0.001).

**Table 2:** Prognostic factors for overall survival

	Univariate analysis (log-rank test)		Multivariate analysis (Cox hazard model)	
Prognostic factors	HR (95 % CI)	*p*	HR (95 % CI)	*p*
Age group	4.2 (3.1–5.6)	<0.001	3.6 (2.1–6.2)	<0.001
Low DLCO^a^	5.2 (3.2–8.7)	<0.001	3.8 (2.1–7.1)	<0.001
IPF^b^	13.8 (7.6–25.0)	<0.001	4.8 (2.1–10.8)	<0.001
Male	2.6 (1.7–3.9)	<0.001	3.2 (1.4–7.1)	0.005
Pulmonary Tbc.^c^	1.5 (1.1–2.2)	0.039		-
Hypertension	1.39 (1.1–1.9)	0.036		-
Diabetes mellitus	1.9 (1.3–2.8)	<0.001		-
Decreased kidney function^d^	2.7 (1.1–7.2)	0.043		-
Stage IB	1.9 (1.4–2.6)	<0.001		-
Lobectomy/bi-lobectomy	1.7 (1.1–2.8)	0.033		-
Thoracotomy	2.8 (2.0–3.9)	<0.001		-

^a^Expected diffusing capacity for carbon monoxide less than 70 % of prediction

^b^Interstitial pulmonary fibrosis

^c^Previous history of pulmonary tuberculosis

^d^Preoperative creatinine level higher than 1.5 mg/dL

Disease-free survival showed unexpectedly discouraging results for the elderly group (5-year disease-free survival rate; 53.3 vs. 80.2 %, *p* < 0.001). In the multivariate analysis, independent prognostic factors for disease-free survival were elderly age (HR = 2.4, *p* < 0.001), male gender (HR = 1.7, *p* = 0.017), IPF (HR = 4.0, *p* < 0.001), low DLCO (HR = 2.0, *p* = 0.006), and stage IB (HR = 2.1, *p* < 0.001). In the subgroup analysis, independent prognostic factors for disease-free survival in elderly patients were male gender (HR = 2.6, *p* = 0.007) and low DLCO (HR = 2.1, *p* = 0.03). Incidence of recurrence within 2 years was also significantly higher in elderly group than in younger group (13.7 vs. 5.7 %, *p* < 0.001). Independent risk factors for recurrence were elderly age (HR = 3.4, *p* = 0.001) and pathologic stage IB over IA (HR = 4.3, *p* < 0.001). Short- and long-term outcomes of stage I lung cancer according to the age group were summarized in Table 3, and results of multivariate analysis in elderly group were also demonstrated in Table 4.

**Table 3:** Short- and long-term outcomes of stage I non-small cell lung cancer

	Elderly group (*n* = 285)	Younger group (*n* = 1055)	*p*
Short-term results			
30-day mortality, *n* (%)	6 (2.2)	0	0.014*
90-day mortality, *n* (%)	11 (3.9)	5 (0.5)	<0.001*
Complication rate, %	47.7	26.9	<0.001*
Hospital stay, days	11.2 ± 12.2	8.0 ± 8.5	<0.001**
Long-term results			
5-year overall survival rate, %	69.0	91.1	<0.001***
5-year disease-free survival rate, %	53.3	80.2	<0.001***
Recurrence rate within 2 years, %	13.7	5.7	<0.001*

*p value estimated by Pearson’s chi-square test: **p value estimated by Student’s *t* test 2; ***p value estimated by the log-rank test

**Table 4:** Multivariate analyses of risk and prognostic factors for short- and long-term results in elderly patients

	Predictive factors	OR/HR (95 % CI)	*p*
Short-term outcomes			
30-day mortality	DLCO less than 70 % of prediction	17.1 (1.46–199)	0.024
90-day mortality	DLCO less than 70 % of prediction	8.47 (1.11–64.6)	0.039
Complication rate	Male gender	7.80 (3.10–19.6)	<0.001
	DLCO less than 70 % of prediction	3.51 (1.03–12.0)	0.045
Hospital stay	Open thoracotomy	6.75 (2.41–11.1)	0.003
	DLCO less than 70 % of prediction	8.64 (1.88–15.4)	0.013
Long-term outcomes			
5-year overall survival	Squamous cell carcinoma	3.18 (1.56–6.48)	0.001
	DLCO less than 70 % of prediction	2.37 (1.10–5.14)	0.028
5-year disease-free survival	Male gender	2.57 (1.29–5.09)	0.007
	DLCO less than 70 % of prediction	2.07 (1.07–4.00)	0.030
Recurrence rate within 2 years	Stage IB	1.7 (1.04–2.75)	0.035

DLCO: expected diffusing capacity for carbon monoxide

OR: odd ratio from logistic regression model for short-term outcomes

HR: hazard ratio from Cox proportional hazard model for long-term outcomes

95 % CI: 95 % confidential interval

To rule out the effect of functional and pathologic differences other than age between two groups, further pair matching analysis was performed for this unexpected high incidence of recurrence rate in elderly patients. Pair matching analysis was performed by propensity score matching, and 285 pairs of elderly and younger patients were matched 1:1. The clinical variables used for matching were gender, comorbidities, preoperative labs, and pulmonary function tests. Pathologic variables were also matched and included cancer stage and histologic subtype. The surgical variables included extent of pulmonary resection and type of operation.

Analysis of the cumulative recurrence rate after pulmonary resection between the elderly and the matched younger controls to evaluate lung cancer-specific long-term outcome still showed discouraging results for the elderly group. Five-year overall survival rates in the unmatched younger, matched younger, and elderly groups were 91.1, 89.5, and 69.0 %, respectively. Five-year disease-free survival rates in the younger and elderly groups were 80.2 %, 75.0 % and 53.3 %, respectively. Three-year cumulative recurrence rates of the younger and elderly groups were 16.0 and 23.2 %, respectively (*p* = 0.001), and stage IB (HR = 1.7, *p* = 0.035) was the only independent risk factor in elderly patients. However, there was no significant difference between the younger group and the elderly group in loco-regional recurrence rate (11.0 vs. 11.3 %, *p* = 0.586). Only distant recurrence rate was significantly different between the younger group and the elderly group (5.6 vs. 13.4 %, *p* < 0.001). The most common extra-thoracic sites of distant recurrence were brain (*n* = 15), liver (*n* = 10), and bone (*n* = 10), for which the incidence of metastasis was significantly higher in the elderly group (3.5 vs. 8.8 %, *p* = 0.009). Comparison of elderly patients and matched younger control was demonstrated in supplementary data.

## Discussion

Several large population studies have examined early results after pulmonary resection in elderly patients and reported that the early mortality among elderly patients was significantly greater [8–10]. In a recent study based on a large French national database performed by Rivera et al., postoperative mortality was significantly higher in elderly patients at every end point compared with younger controls (30-day mortality; 3.6 vs. 2.2 %, *p* = 0.010, 90-day mortality; 4.7 vs. 2.5 %, *p* < 0.001), and the authors pointed out that the 90-day mortality rate seems to better represent real mortality risk than the 30-day mortality rate [11], which was supported by our data (30-day mortality; *p* = 0.014, 90-day mortality; *p* < 0.001, Table 3). On the other hand, Cerfolio and colleagues reported in their nested case-control study that there was no significant difference in 30-day or in-hospital mortality between younger and elderly patients (3.8 vs. 2.2 %, *p* = 0.20) [12]. However, the proportion of male patients was significantly lower in their elderly group after matching, and other comorbidities that might be associated with early outcome in elderly patients were not included as matching variables. Recently, Rueth et al. reported that male gender, higher comorbidity index and age older than 75 years significantly increased postoperative mortality [13]. These discouraging results of elderly group might have been caused by the fragility involved with aging process. Fragility, which is often defined as unintentional weight loss, self-reported exhaustion, muscular weakness, slow walking speed, and low physical activity, is identified as an independent risk factor for major morbidity, mortality, protracted length of stay, and institutional discharge [14, 15].

To compare long-term outcome, Sigel et al. analyzed 27,859 cases of stage I NSCLC from surveillance, epidemiology, and end results (SEER) data and showed that the overall 5-year survival rate declined slightly with increasing age in male patients who underwent surgical resection (age groups <60, 61–69, 70–79, ≥80; 69.2, 66.0, 62.8, and 63.5 %, respectively) but remained similar across all ages [16]. Cerfolio and colleagues reported that 5-year overall survival in their elderly group (≥70 years) was 78 %, which was surprisingly better than the rate in their younger group (69 %, *p* = 0.01) with stage I NSCLC [12]. Nonetheless, generally speaking, chronological age itself is still considered a major risk factor in long-term follow-up. In a study based on SEER conducted by Mery et al. [17] and retrospective matched study by Goodgame et al. [18], both authors reported worse overall survival for elderly patients.

Few studies have examined the association between lung cancer recurrence and age. Maeda et al. analyzed risk factors for the recurrence of stage I NSCLC; finding that old age (≥65 years) was a risk factor with borderline significance for cancer recurrence in a multivariate analysis (*p* = 0.051) [19]. In contrast, Goodgame and colleagues reported that cancer recurrence rate in elderly patients was the same as that in younger patients (3-year cumulative recurrence rate; 19 vs. 20 %, *p* = 0.425) [18]. In our study, the recurrence rate in elderly patients was significantly higher than in younger patients (recurrence rate within 2 years; 13.7 vs. 5.7 %, *p* < 0.001). This finding suggests that lung cancer in elderly patients is not always less virulent. Another explanation for more frequent distant failure in elderly patients might be less aggressive mediastinal lymph node dissection. Recently, Wang et al. reported that elderly patients without radical mediastinal lymphadenectomy experienced more frequent distant relapse compared to those who did undergo the procedure [20]. However, as shown in this study with systemic lymph node dissection in any age group, the cancer-free survival in the elderly was lower than in the younger patients. Therefore, more frequent and meticulous follow-up should be needed in the elderly group.

**Fig. 2:**
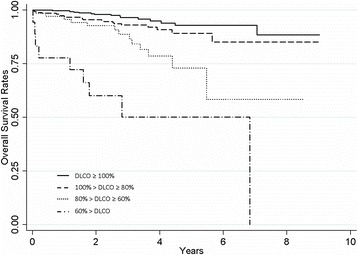
Overall survival according to the level of diffusion capacity. Five-year overall survival rates in stage I lung cancer were significantly associated to the level diffusion capacity with hazard ratio of 0.11 (95 % CI, 0.05–0.24) in the group with DLCO less than 80 % and with hazard ratio of 0.06 (95 % CI, 0.02–0.13) in the group with DLCO less than 60 %

In this study, early and long-term outcomes in elderly patients were worse than expected, and decreased DLCO was the common predictive factor for poor results both in early and long-term outcomes. The importance of DLCO has been recognized in many gerontologic studies. Yano et al. reported that old age (≥70 years), pneumonectomy, and DLCO less than 70 % of predicted significantly increased life-threatening morbidity after pulmonary resection for lung cancer [21]. Ferguson et al. studied patients who underwent surgery for lung cancer and showed that the incidence of postoperative complications doubled in case of low diffusion capacity (DLCO less than 80 % of predicted) [22–24]. For long-term outcome, it is not fully understood whether low diffusion capacity affects prognosis. Ferguson et al., in their study mentioned above, reported that low FEV_1_ did not affect long-term prognosis, but DLCO less than 60 % of predicted significantly worsened overall survival [24]. Another study reported that a DLCO less than 40 % best predicted decreased survival from causes other than cancer within stage I lung cancers [25]. Therefore, surgery for elderly patients with low diffusion capacity may require extra caution or reconsideration of alternative treatments, including limited resection or radiotherapy [26–28]. In this study, patients with DLCO less than 80 % or less than 60 % showed significantly worse outcome in overall survival (HR = 4.6, *p* < 0.001 and HR = 17.9, *p* < 0.001, respectively). Overall survival according to DLCO is presented in Fig. 2. According to the results of this study, pulmonary resection for lung cancer in elderly patients was far more risky than in younger patients. It is difficult to point out the risks of being old, but we presume that it comes from complex reasons, such as fragility to postsurgical stress and life expectancy of belonging society, and a searching analysis of the elderly patients are required in future studies. Until then, elderly patients who undergo pulmonary resection for lung cancer might require more rigorous application of operability than younger patients. Especially with the patients with decreased DLCO, additional assessments such as exercise pulmonary function test should be performed prior to surgery, and if the patients are judged to be unsuitable for surgery, alternative treatments should be considered. For patients who are medically inoperable for early lung cancer, stereotactic body radiation therapy (SBRT) has been widely applied and reported to be highly effective at controlling the primary tumor without serious toxicity. Radiation Therapy Oncology Group 0236 trial, which enrolled inoperable 59 patients with T1-2 lung cancer, showed 3-year survival of 55.8 % after SBRT and high rates of local tumor control (3-year primary tumor control rate of 97.6 %.) [29]. Onishi et al. reported even better results, and the 5-year overall survival rates in this study showed 72 and 62 % for stage IA and IB, respectively [30].

This study was a retrospective study and has associated limitations. Potential confounders affecting results after pulmonary resection in younger and elderly groups were minimized by matching clinicopathologic variables and surgical factors. Another limitation is that other known independent factors from previous studies, such as preoperative serum carcinoembryonic antigen level or lymphovascular invasion in pathologic reports, were not evaluated due to incomplete availability of these parameters in our database [31, 32]. Also, socioeconomic position of patients, which is also another important predictive factor in cancer treatment, was not considered in this study [33].

## Conclusions

In conclusion, elderly patients with stage I NSCLC showed unsatisfying results in both short- and long-term outcomes. Especially, elderly patients with low DLCO are at risk for significantly worse outcome, indicating that patient selection should include assessment of pulmonary function, including DLCO, and also, more frequent following up should be needed.

## Abbreviations

NSCLC: non-small cell lung cancer
FEV_1_: forced expiratory volume in 1 second
FVC: forced vital capacity
DLCO: diffusing capacity for carbon monoxide
ARDS: acute respiratory distress syndrome
OR: odd ratio
HR: hazard ratio
IPF: interstitial pulmonary fibrosis
SEER: surveillance, epidemiology, and end results
